# Ginsenoside Rg3 treats acute radiation proctitis through the TLR4/MyD88/NF-κB pathway and regulation of intestinal flora

**DOI:** 10.3389/fcimb.2022.1028576

**Published:** 2023-01-06

**Authors:** Xiaoyu Duan, Hongyi Cai, Tingting Hu, Lili Lin, Lu Zeng, Huixia Wang, Lei Cao, Xuxia Li

**Affiliations:** ^1^ The First Clinical Medical College of Gansu University of Chinese Medicine (Gansu Provincial Hospital), Lanzhou, China; ^2^ Department of Radiotherapy, Gansu Provincial Hospital, Lanzhou, China

**Keywords:** TLR4/MyD88/NF-κB signal pathway, ginsenoside Rg3, acute radiation proctitis, intestinal microflora, mechanism

## Abstract

**Objectives:**

This study aimed to investigate the protective effect of ginsenoside Rg3 (GRg3) against acute radiation proctitis (ARP) in rats.

**Methods:**

Wistar rats were randomly divided into control, model, dexamethasone-positive, GRg3 low-dose, GRg3 medium-dose, and GRg3 high-dose groups. The ARP rat model was established by a single 22-Gy irradiation of 6 MV) X-rays. The distribution and function of intestinal flora were detected using 16S rRNA high-throughput sequencing, rectal tissue was observed by hematoxylin and eosin (H&E) staining, the expression of interleukin 1β (IL-1β) and IL-10 inflammatory factors was detected by ELISA, and mRNA and protein expression of toll-like receptor 4 (TLR4), myeloid differentiation primary response 88 (MyD88), and nuclear factor kappa-light-chain-enhancer of activated B cells (NF-κB) were detected by RT-qPCR and Western blotting, respectively.

**Results:**

GRg3 improved the symptoms of ARP in rats in a dose-dependent manner. The species distribution of intestinal flora in GRg3 rats was significantly different from that in ARP rats. These differences were more significant in the high-dose group, where the numbers of *Ruminococcus*, *Lactobacillus*, and other beneficial bacteria were significantly increased, whereas those of *Escherichia*, *Alloprevotella*, and other harmful bacteria were decreased. In addition, GRg3 was closely related to amino acid metabolism. After GRg3 treatment, the mRNA and protein expression of TLR4, MyD88, and NF-κB in rectal tissue was significantly down-regulated, and the level of downstream inflammatory factor IL-1β decreased, whereas that of IL-10 increased.

**Conclusion:**

Our study indicated GRg3 as a new compound for the treatment of ARP by inhibiting the TLR4/MyD88/NF-κB pathway, down-regulating the expression of proinflammatory factors, thus effectively regulating intestinal flora and reducing inflammatory reactions.

## 1 Introduction

Radiotherapy is an effective treatment for malignant abdominal, retroperitoneal, and pelvic tumors. In recent years, with the continuous progress in radiotherapy technology, ionizing radiation (IR) can be used to accurately identify targeted tumors; however, it can also damage normal intestinal tissue, resulting in radiation enteritis (RE) (Wang et al., 2021). Owing to the anatomic position of the rectum and the dose-dependent toxicity of IR, rectal tissue is easily damaged. After IR, approximately 50%–75% of patients develop acute radiation proctitis (ARP) within 3 months, with 20% of them gradually developing chronic radiation proctitis (CRP) (Grodsky and Sidani, 2015). At present, the molecular mechanisms underlying the development of ARP are not well understood.

As a pattern recognition receptor, toll-like receptor 4 (TLR4) triggers acute inflammation responses immediately following the recognition of invading bacteria, fungi, viruses, and other microorganisms. The mechanism by which the TLR4 pathway promotes the inflammatory response against pathogens involves the combined action of bacterial lipopolysaccharides and binding proteins that directly activate TLR4 under the transport of CD14 in immune cells. This triggers the signal cascade of toll/interleukin-1 receptor domain-containing adapter protein (TIRAP) and myeloid differentiation primary response 88 (MyD88) binding proteins located on the plasma membrane, further activating the nuclear translocation of NF-κB transcription factors, thus promoting the expression of inflammatory factors (Liu et al., 2021). Some studies have found that TLR4 promotes RE by recognizing high-mobility group box 1 protein (HMGB1) and NF-κB and activating inflammatory factors, such as interleukin 1β (IL-1β), tumor necrosis factor alpha (TNF-α), and IL-6 (Zhao et al., 2020; Zhang et al., 2020). The TLR4 pathway participates in the inflammatory response in many diseases; however, its effect on ARP has not been documented.

The intestinal flora maintains a dynamic balance in the body and plays a key role in metabolism, nutrition, physiology, and immunity (McKenzie et al., 2017). Changes in the internal and external environment disrupt this balance and alter the proportion, number, species, and metabolites of intestinal flora, resulting in intestinal flora imbalance. Tight junction proteins are the most important guardians of the intestinal barrier. Studies have shown that intestinal flora imbalance is mediated by down-regulating the expression of tight junction proteins, destroying the function of the intestinal epithelial barrier, promoting the expression of inflammatory factors, and hence aggravating radiation enteritis (Stringer, 2013; Zihni et al., 2016; Jian et al., 2021). The abundance and function of intestinal flora species are also changed in patients with CRP (Liu et al., 2021). Therefore, intestinal flora imbalance might serve as a biomarker for the diagnosis and prognosis prediction of ARP.

Ginsenoside Rg3 (GRg3), which is the main active component of *Panax ginseng*, has anti-inflammatory, antitumor, antiangiogenic, and immune-enhancing effects. Some studies have shown that GRg3 significantly reduces the expression of the IL-6, TNF-α, and cyclooxygenase-2 (COX-2) proinflammatory factors in airway epithelial cells of asthmatic mice, thereby reducing airway inflammation (Huang et al., 2021). In addition, GRg3 has been reported to be involved in the regulation of the activation of the phosphoinositide 3-kinase (PI3K)/protein kinase B (PKB, also known as AKT)/mammalian target of rapamycin (mTOR) and inhibition of the NF-κB signaling pathways and has, therefore, been widely used in the treatment of acute lung injury (Cheng and Li, 2016; Lee et al., 2016; Yang et al., 2018). However, despite being a good anti-inflammatory drug, the effect of GRg3 on the IR-induced inflammatory response has not been studied yet. We previously demonstrated that GRg3 exhibits a good anti-inflammatory effect in ARP rats (Hu and Cai, 2019). In this study, we investigate the related underlying mechanism of GRg3 in the treatment of ARP.

## 2 Materials and methods

### 2.1 Experimental animals and reagents

Forty-eight male Wistar rats of SPF grade, weighing 180–200 g, were purchased from Beijing Spelford Biological Company (license number: SCXK (Beijing) 2019-0010; Beijing, China). Rats were raised in the SPF laboratory of the Experimental Animal Center of Lanzhou University under steady conditions of temperature at 20–26°C, 45–55% humidity, and a 12/12 h light and dark cycle, with free access to food and water. This study strictly complied with the care and use of laboratory animals in the People’s Republic of China (revised in 2006) and was approved by the Medical Ethics Committee of Gansu Provincial People’s Hospital (approval number: 2021-287). Related reagents’ information was shown in supplementary materials.

### 2.2 Establishment of animal model

Forty-eight rats were fed adaptively for 1 week and then randomly divided into six groups, with eight rats in each group: control check (CK), model (MG), dexamethasone-positive (Dexa), GRg3 low-dose (GL), GRg3 medium-dose (GM), and GRg3 high-dose (GH). Rats in all groups except for the CK group, were fasted and were not allowed water for 12 h before irradiation. Rats were anesthetized with pentobarbital sodium (30 mg/kg, intraperitoneal injection). After anesthesia, the local area of each rat was irradiated with 22 Gy using a 6-MV X-ray beam. The irradiation range covered the area from the pubic symphysis to the anus, which was surrounded by a lead plate. The radiation field area was 3.5 cm × 5 cm, the source skin distance was 100 cm, and the dose rate was 300 cGy/min. Changes in the fecal morphology (diarrhea, mucinous or loose stools) of rats within 1–7 days after irradiation were considered indicative of the establishment of a successful model. Rats were subjected to continuous intragastric administration of the experimental compounds for 14 days, starting from the eighth day. The CK and MG groups were given the same amount of saline and the Dexa group was treated with 1.425 mg/kg dexamethasone sodium phosphate, while the GL, GM, and GH groups were perfused with 20, 40, and 80 mg/kg GRg3, respectively.

### 2.3 Disease activity index

The mental state and food and water intake of rats were observed, their body weight and stool morphology were recorded, and disease activity was evaluated daily. The disease activity index (DAI) was scored according to the percentage of weight loss, fecal characteristics, and fecal bleeding, and the total score of the three combined results was divided by 3. Scoring criteria are presented in **Supplementary Table 1**.

### 2.4 16s rDNA sequencing and data analysis

Fresh rat feces were collected, and DNA was extracted. A small fragment library was constructed based on the amplified 16s region (V3–V4 region), and double-terminal sequencing was performed using the Illumina NovaSeq sequencing platform. The sequenced data were spliced and subjected to quality control, chimera filtering, and operational taxonomic unit (OTU) clustering. Based on the above data, the species distribution of intestinal flora was analyzed, differences in species composition and community structure among samples were revealed, and the function of related genes was predicted.

### 2.5 Hematoxylin–eosin staining

Following fixation with 4% paraformaldehyde, rat rectal tissues were pruned, dehydrated, embedded, sliced, and stained with H&E. Finally, sections were observed under a microscope.

### 2.6 Enzyme-linked immunosorbent assay

Blood was collected from the abdominal aorta, centrifuged at 3,000 rpm for 20 min, and the supernatant was collected. The levels of the proinflammatory factor IL-1β and anti-inflammatory factor IL-10 in the sera of rats in each group were detected using the appropriate ELISA kits and in accordance with the manufacturer’s instructions.

### 2.7 Real-time quantitative PCR

Total tissue RNA was extracted using the TRIzol reagent and reverse transcribed into cDNA using the reverse transcription kit. SYBR Green Master Mix was used for PCR amplification to detect the relative expression of *TLR4*, *MyD88*, and *NF-κB* mRNAs in rectal tissues. PCR conditions were as follows: predenaturation at 95°C for 5 min, followed by 40 cycles of denaturation at 95°C for 10 s, annealing at 55°C for 20 s, and extension at 72°C for 20 s. Primer sequences are listed in **Supplementary Table 2**.

### 2.8 Western blotting

Rectal tissue was added to 1 ml of radioimmunoprecipitation assay (RIPA) lysate and 10 μl of phenylmethylsulfonyl fluoride (PMSF) at ratios of 1:10 and 1:100, respectively. The NF-κB p65 protein was cleaved by the addition of 10 μl of phosphorylated protease inhibitor A and B solution. Protein concentration was determined using the bicinchoninic acid assay (BCA) method. Equal amounts of protein were separated by SDS-PAGE and then transferred to polyvinylidene fluoride (PVDF) membranes. Membranes were then incubated with primary antibodies at 4°C overnight, followed by an incubation with secondary antibodies (1:5,000) for 1 h at 25°C, and finally developed using an enhanced chemiluminescence (ECL) chemiluminescent solution. The dilution ratio of TLR4, MyD88, and NF-κB P65 antibodies was 1:1,000, whereas that of the β-actin antibody (internal control) was 1:2,000.

### 2.9 Statistical analysis

SPSS 25.0 and GraphPad Prism 9 were used for statistical analysis and mapping. Measurement data were expressed as the mean ± standard error ( ± s). The Student’s *t*-test was used for comparisons between the two groups. One-way analysis of variance (ANOVA) was used for comparisons between multiple groups, and the Bonferroni test was used for *post hoc* analysis. A *p*-value of < 0.05 was considered statistically significant.

## 3 Results

### 3.1 GRg3 effectively improved the general condition of ARP rats

**Figure 1:**
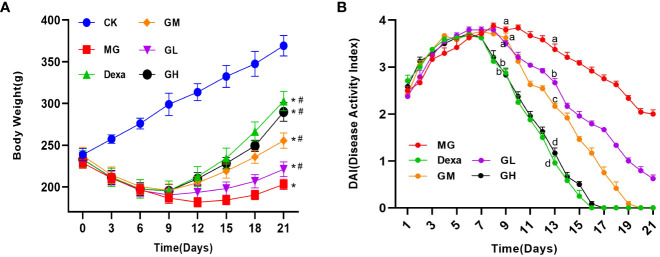
The general condition of rats in each group (rats received continuous intragastric treatment for 14 days after irradiation, i.e., from day 8 to day 21). **(A)** Change in rat body weight compared with the CK group (^*^ *p *< 0.05) and the MG group (^#^ *p*<0.05). **(B)** Rat DAI. A letter common to two groups indicates a significant difference (*p*>0.05). The absence of a common letter between two groups indicates a *p-*value of <0.05.

We observed that ARP rats gradually showed signs of listlessness, slow activity, reduced water intake, loss of lower abdominal or perianal hair, diarrhea, and blood in the stool on the second day after irradiation. From the eighth day after irradiation, we started to detect differences in the body weight and DAI of rats among treatment groups. On day 9, we found that the DAI was significantly decreased in the Dexa and GH groups (*p* < 0.0001), whereas we did not observe any significant differences in the DAI of the GL and GM groups (*p* > 0.05), compared with that in the MG group. On day 13, we found that the DAI was lower in all treatment groups than in the MG group (*p* < 0.05). On day 21, the body mass of rats in the treatment groups was significantly higher than that in the MG group (*p* < 0.05). Interestingly, we noticed that the DAI in the GM, GH, and Dexa groups was 0. These results suggest that dexamethasone and high-dose ginsenoside Rg3 significantly improved the symptoms of diarrhea and hematochezia in ARP rats in a short time, whereas medium-dose ginsenoside Rg3 gradually alleviated the symptoms of ARP with an extension of treatment time; however, the therapeutic effect of low-dose ginsenoside Rg3 was minimal (**Figures 1A, B**).

### 3.2 GRg3 regulated the composition of intestinal flora in ARP rats

#### 3.2.1 Flora identification and species distribution

We found that, at the phylum level, the abundance of *Firmicutes* and the *Firmicutes* to *Bacteroidota* were lower in the MG group than in the CK group, whereas the abundance of *Proteobacteria* was higher in the MG than in the CK group. We also found that, compared with the MG group, the abundance of *Firmicutes* was higher, whereas that of *Proteobacteria* was lower, in the Dexa and GH groups.

At the family level, we identified that, compared with the CK group, the abundances of *Clostridiaceae* and *Enterobacteriaceae* were increased, whereas those of *Lachnospiraceae* and *Peptostreptococcaceae* were decreased in the MG group. In addition, we found that the abundances of *Prevotellaceae* and *Enterobacteriaceae* were decreased, whereas those of *Peptostreptococcaceae* and *Lactobacillaceae* were increased, in the GH group compared with the MG group.

**Figure 2:**
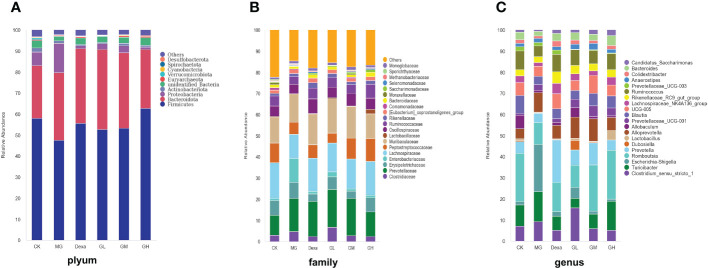
Relative abundance bar chart of species. The species abundance maps of **(A)** phyla, **(B)** families, and **(C)** genera are shown.

Finally, we found that, at the genus level, the abundances of *Escherichia* and *Shigella* were increased, whereas those of *Romboutsia*, *Bacteroides*, *Ruminococcus*, *Lachnospiraceae* NK4A136, and *Blautia* were decreased in the MG group compared with the CK group. Interestingly, we further identified that, compared with the MG group, the abundances of *Escherichia*, *Shigella*, and *Alloprevotella* were decreased in the Dexa and GH groups, whereas the abundances of *Romboutsia*, *Blautia*, *Lachnospiraceae* NK4A136, and *Ruminococcus* were increased in the GM and GH groups (**Figures 2A–C**).

#### 3.2.2 Alpha and beta diversity indices

We selected seven different alpha diversity indices and used the Wilcoxon test to assess the diversity, evenness, sequencing depth, and richness of species distribution between two groups. Our results on alpha diversity revealed that the number, richness, diversity, homogeneity, and genetic relationship of species were significantly lower in the MG group than in the CK group (*p* < 0.05). However, we did not observe any significant differences in species number and richness between the MG and GH groups (*p* < 0.05). We also noticed that species richness was higher in the GH group than in the GM group (*p* < 0.05). Finally, we did not detect any statistically significant differences in the sequencing depth among all groups (*p* > 0.05) (**Supplementary Figure 1A–G**).

Using the principal coordinate analysis (PCoA) beta diversity index, we identified significant differences in the characteristics of intestinal flora between the CK and GH groups and between the MG and GH groups (*p* < 0.05). Interestingly, we found that the characteristics of intestinal flora were similar among the other groups. This finding indicates that IR affected the distribution of intestinal flora in ARP rats, and only a high dose of GRg3 could alter the intestinal flora (**Supplementary Figure 1H**).

#### 3.2.3 Analysis of microflora differences among groups

**Figure 3:**
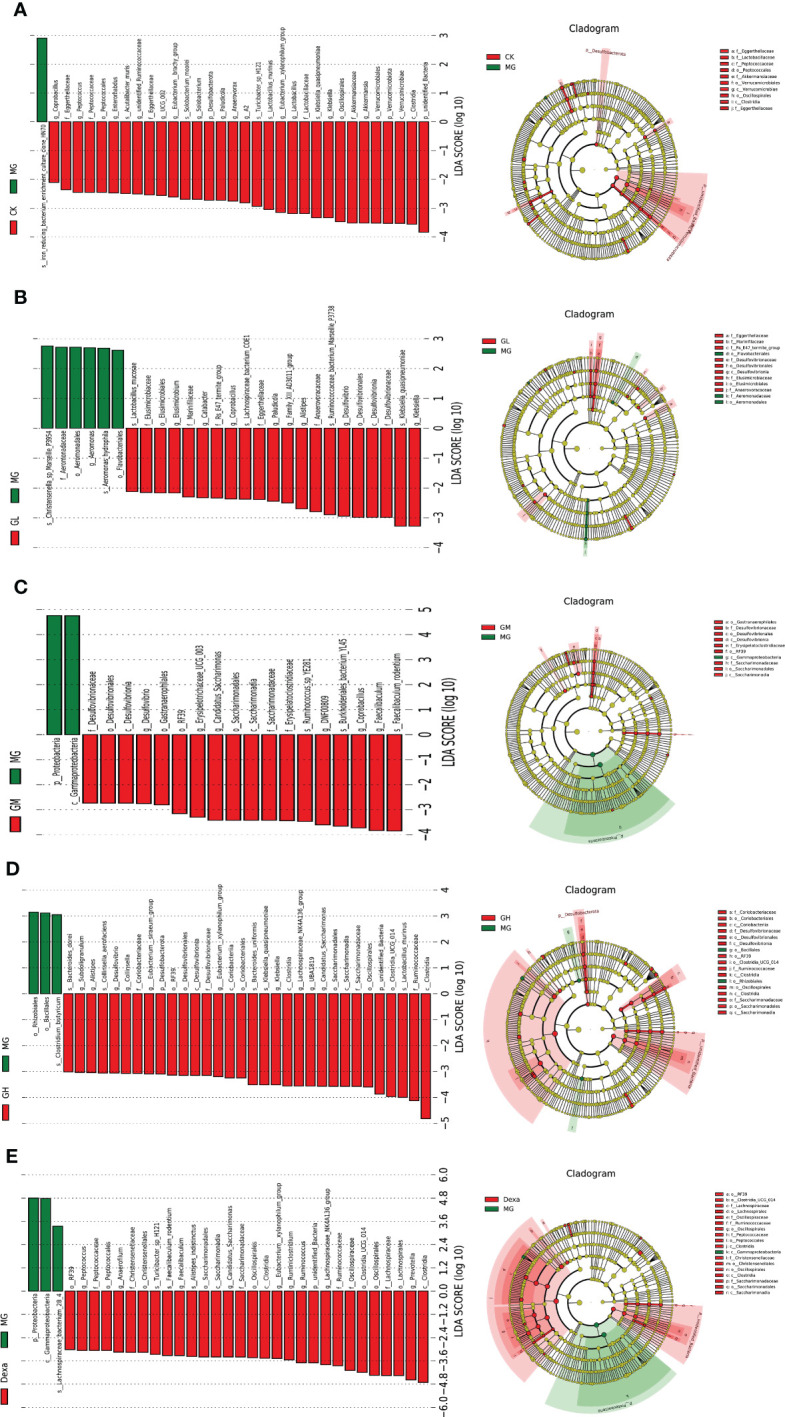
Left: histogram of linear discriminant analysis (LDA) value distribution (LDA>2); right: the evolutionary cladogram of LEfSe. Differential flora between two groups: CK and MG groups **(A)**, GL and MG groups **(B)**, GM and MG groups **(C)**, GH and MG groups **(D)**, Dexa and MG groups **(E)**.

We then used the linear discriminant analysis effect size (LEfSe) to screen for differential bacteria between two groups. When comparing the CK and MG groups, we detected the presence of an iron-reducing bacterial culture clone, termed *hn70*, at the family level, which was highly abundant in the MG group. In our comparison between the GL and MG groups, we found that *Flavobacteriales*, *Aeromonadaceae*, and *Aeromonas hydrophila* were enriched in the MG group, whereas *Klebsiella* and *Quasipneumoniae* were the dominant flora in GL group. Comparison between the GM and MG groups revealed that *Coprobacillus* and *Faecalibaculum rodentium* were the predominant bacteria in the GM group. When comparing the GH and MG groups, we found that *Clostridia*, *Ruminococcaceae*, and *Lactobacillus murinus* were enriched in the GH group, while in the comparison of the Dexa and MG groups, we observed that *Clostridia* and *Prevotella* were the predominant bacteria in the Dexa group (**Figures 3A–E**).

#### 3.2.4 Species correlation network diagram at the genus level

We obtained the species symbiosis network diagram of GH by calculating the Spearman’s correlation coefficient of all samples. We found that there was a positive correlation between *Turicibacter* and *Romboutsia*, whereas a negative correlation was found between *Turicibacter* and *Lachnospiraceae* NK4A136. We also detected that *Lactobacillus* was positively correlated with *Romboutsia*, but was negatively correlated with *Prevotella*, *Parabacteroides*, *Alloprevotella*, and *Lachnospiraceae* NK4A136. Finally, we observed a negative correlation between *Ruminococcus* and *Prevotella* and between *Clostridium* and *Bacteroides* (**Supplementary Figure 2**).

#### 3.2.5 Predictive analysis of gene function

We then used the KEGG (Kyoto Encyclopedia of Genes and Genomes) database to annotate the functional information of the whole genome of prokaryotes. Our principal coordinate analysis (PCoA) analysis revealed differences in the function of microbiota between the MG group and each treatment group. Interestingly, we observed that microbial functions were similar among different GRg3 dose groups but differed from those in the CK group. The functional relative abundance clustering heat map showed enrichment in cysteine and methionine metabolism in the GH group, whereas transporters were enriched in the MG group. We then analyzed the functions of differential genes in the MG and GH groups using the *t*-test. Thus, we identified significant differences in transporters, exosomes, quorum sensing, alanine aspartate, and glutamate metabolism, among others, between the MG and GH groups (*p* < 0.01) (**Supplementary Figure 3A–C**).

### 3.3 GRg3 altered the pathological manifestations of ARP rats

**Figure 4:**
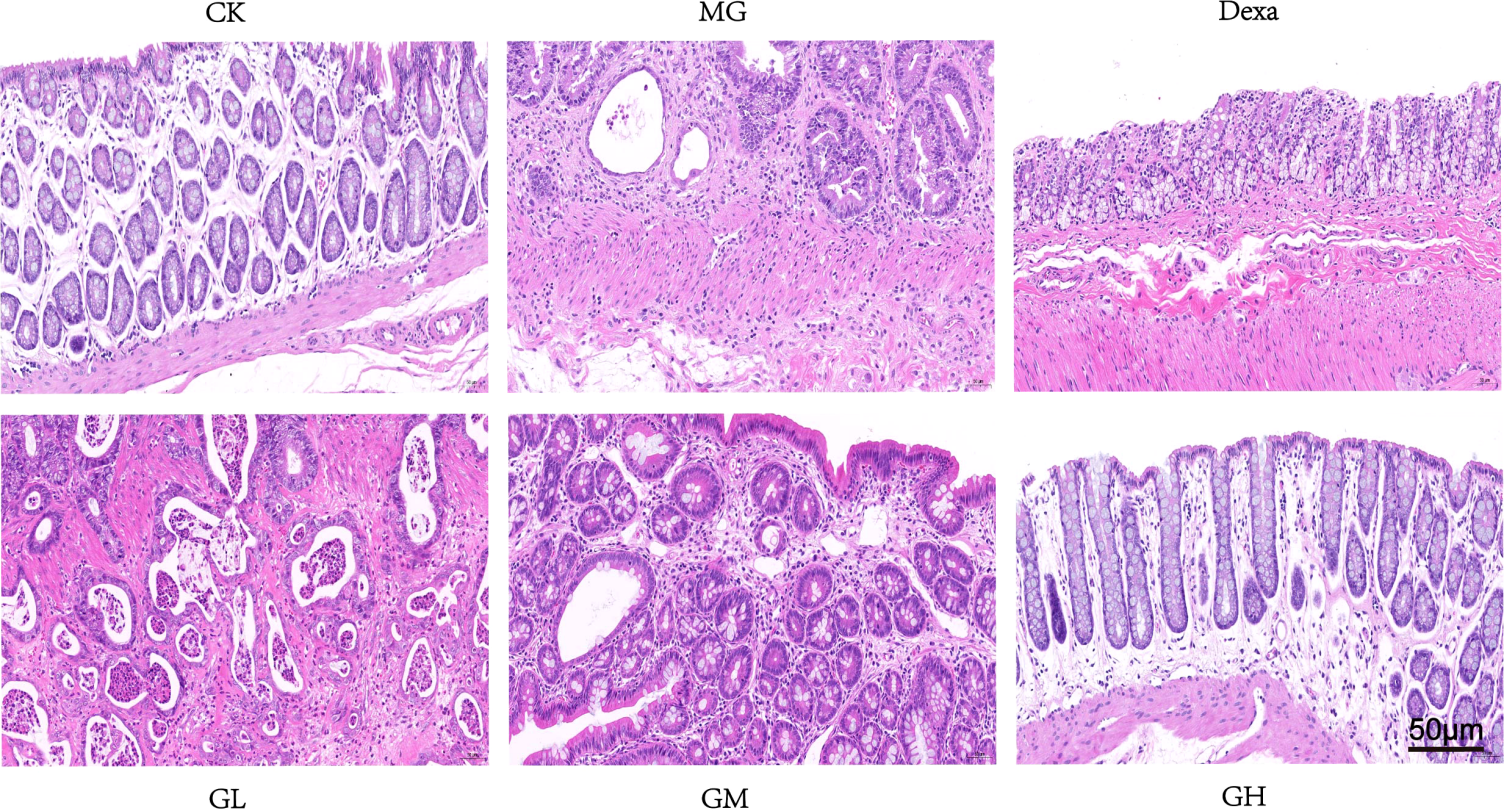
Pathological changes in rectal tissue in each group after 14 days of GRg3 intragastric treatment.

We observed that the intestinal glands were arranged neatly and loosely in the rectal tissue, and the space was slightly widened in rats in the CK groups. However, in the MG group, we detected large-area ulcers in the rectal tissue, invading the serosa and accompanied by a large infiltration of lymphocytes, numerous dilated intestinal glands, necrotic cell fragments in the lumen, severe edema of the submucosa, and uneven thickness of the muscle layer. The rectal glandular lumens of the GL and GM groups had necrotic cell fragments accompanied by a reduced infiltration of inflammatory cells. In the Dexa and GH groups, we detected extensive mucosal edema, loose arrangement of the intestinal glands, widening space, and a slight infiltration of inflammatory cells. Overall, we concluded that GRg3, especially at a high dose, significantly altered the pathological characteristics in MG rats (**Figure 4**).

### 3.4 GRg3 reduced inflammation

**Figure 5:**
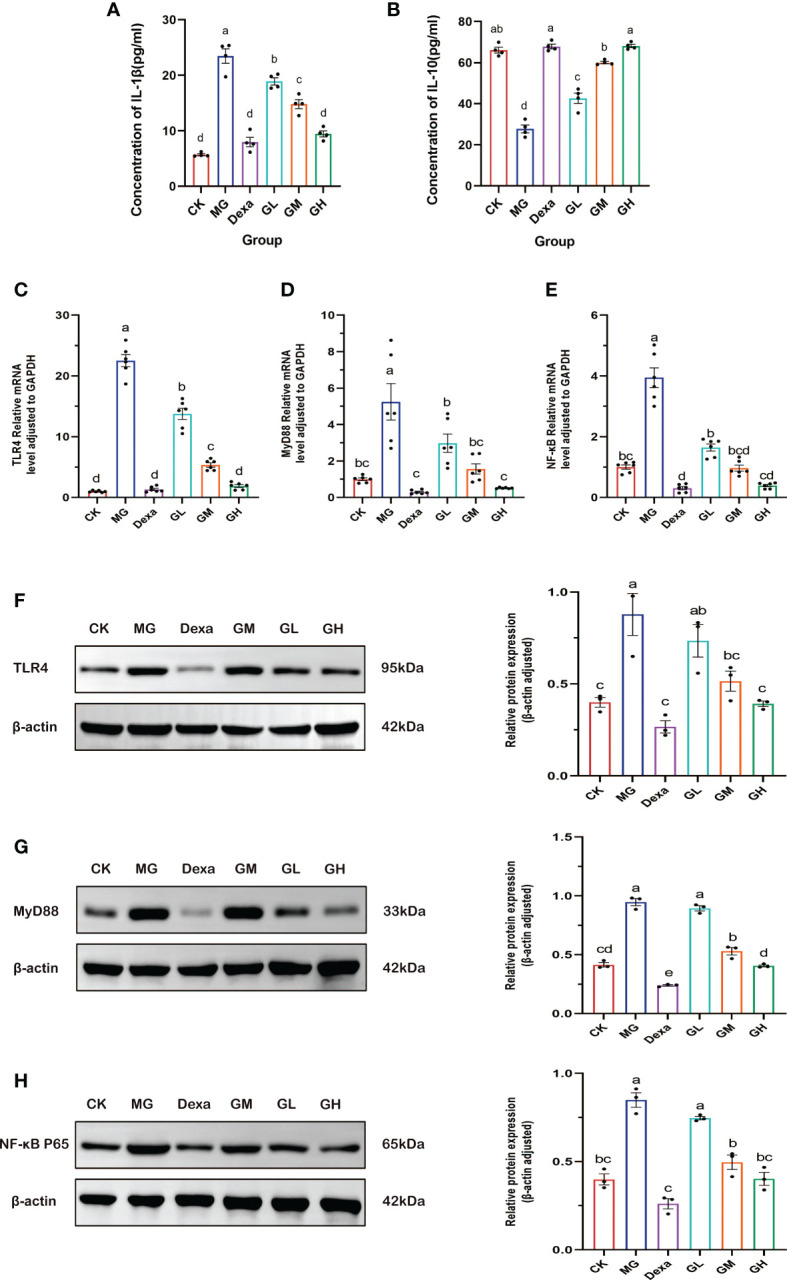
The expression level of inflammatory factors as detected by ELISA. **(A)** IL-1β content. **(B)** IL-10 content. **(C–E)** RT-qPCR was used to detect the relative mRNA expression of the genes encoding *TLR4*, *MyD88*, and *NF-κB*. **(F–H)** WB was used to detect the relative expression of the proteins TLR4, MyD88, and NF- κB. A letter common to two groups indicates a significant difference (*p* > 0.05). The absence of a common letter between two groups indicates a *p*-value of <0.05).

We found that the concentration of IL-1β in sera was significantly higher (*p* < 0.0001), and the content of IL-10 in sera significantly lower (*p* < 0.001), in rats in the MG group than in those in the CK group. Interestingly, the level of IL-1β decreased (*p *< 0.01), whereas that of IL-10 significantly increased (*p* < 0.0001) in all treatment groups compared with levels in the MG group. These results showed that even low doses of GRg3 were effective in suppressing inflammation (**Figures 5A, B**).

### 3.5 GRg3 down-regulated the mRNA and protein expression of TLR4, MyD88, and NF-κB

We evaluated the mRNA expression of the genes encoding *TLR4*, *MyD88*, and *NF-κB* using RT-qPCR. We found that the levels of expression of these three genes were significantly higher in the MG group than in the CK group (*p* < 0.0001).Conversely, the expression of these genes was reduced in all treatment groups compared with those in the MG group (*p* < 0.05), especially in the GH and Dexa groups, which exhibited the most significant down-regulation (*p* < 0.0001) (**Figure 5C–E**).

We then measured expression of TLR4, MyD88, and NF-κB p65 proteins by Western blotting. We observed that, compared with the CK group, the expression of all proteins was increased in the MG group (*p* < 0.001). Of note, compared with the MG group, we did not detect any significant differences in the expression of these three proteins in the GL group (*p* < 0.05), whereas we found that expression of these proteins was decreased in the other treatment groups (*p* < 0.05). The greatest reduction in the levels of these proteins occurred in the GH and Dexa groups (*p* < 0.001) (**Figures 5F–H**).

The above results show that GRg3 significantly restrained the expression of factors involved in the TLR4 pathway in a dose-dependent manner, especially in rats treated with high-dose GRg3.

## 4 Discussion

**Figure 6:**
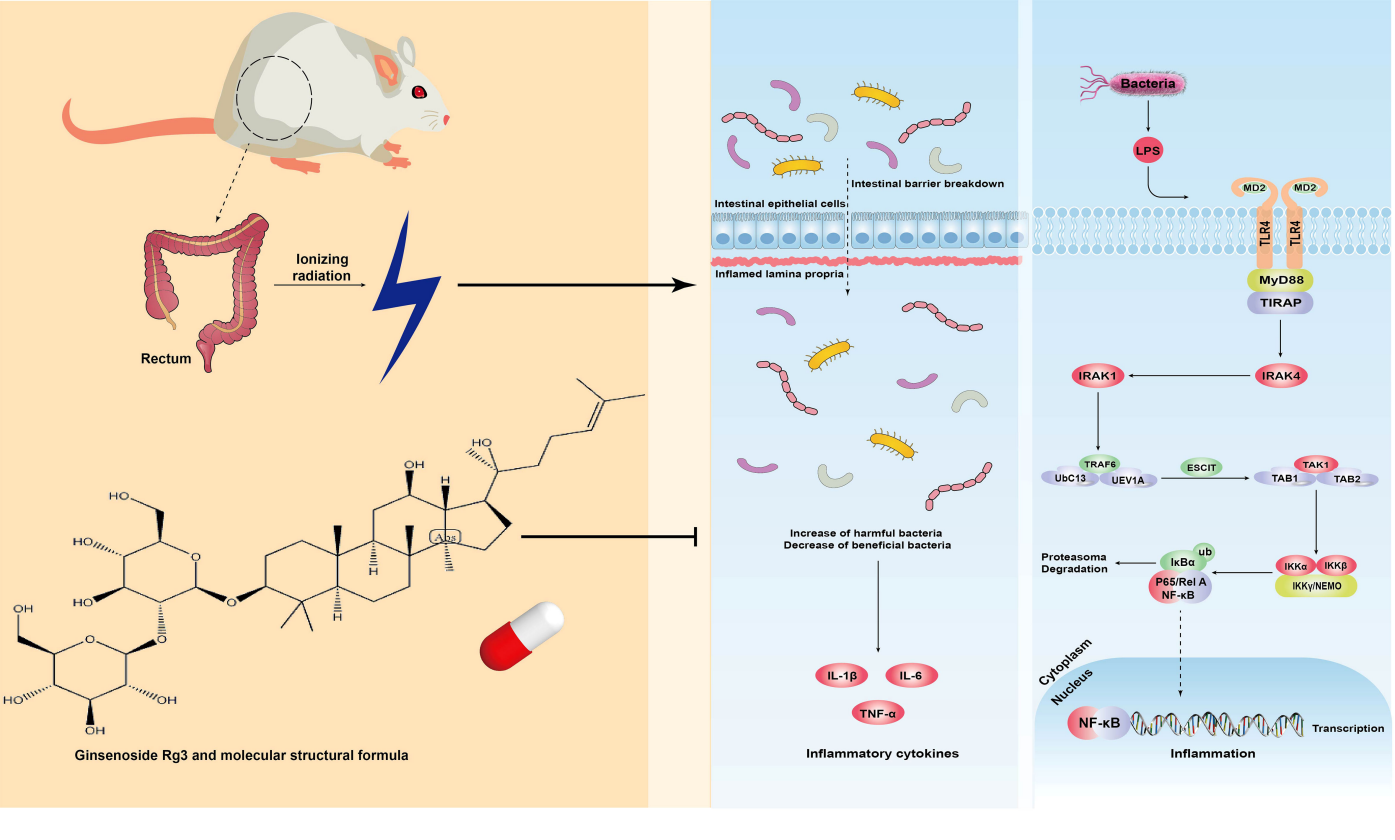
GRg3 mechanism diagram.

According to statistics, approximately 1.5–2 million patients with cancer suffer from radiotherapy-induced intestinal damage, which greatly affects their quality of life (Hauer-Jensen et al., 2007). ARP is one such common complication. The mechanisms of development of ARP include IR-induced DNA double-strand breaks, early intestinal crypt stem cell depression, mucous layer and lamina propria damage, and acute inflammation from infiltrating T lymphocytes, macrophages, and neutrophils following the invasion of pathogens and exposure of the site (Grodsky and Sidani, 2015). Subsequently, a large number of inflammatory metabolites continue to damage submucosal tissues and aggravate intestinal wall injury (Shadad et al., 2013). At present, ARP is mainly treated by the administration of dexamethasone, montmorillonite powder, antibiotics, and aminosalicylic acid; however, these therapies are often associated with the occurrence of adverse reactions, such as rash, muscle and joint pain, and abdominal distension (Hale, 2020). Recently, the administration of widely recognized probiotic preparations was shown to have a certain curative effect, but long-term use will lead to regimen dependence, which is not conducive to the self-reproduction of intestinal flora. Therefore, there is an urgent need for drugs that can target and cure ARP with or without the occurrence of minor side effects. In this study, we investigated the mechanism by which GRg3 reduces inflammation and regulates intestinal flora in ARP rats (**Figure 6**).

### 4.1 The mechanism of the anti-inflammatory effect of GRg3

TLR4 is a member of the toll-like receptor family and is mainly expressed in monocytes, macrophages, dendritic cells, and endothelial cells (Vaure and Liu, 2014). TLR4 phosphorylates the inactive NF-κB in the cytoplasm *via* an MyD88-dependent pathway, thus leading to the production of a large number of inflammatory factors (Lawrence and Fong, 2010; Vajjhala et al., 2017; Tang et al., 2021). NF-κB plays an important role in the occurrence and development of inflammatory bowel disease by inducing the expression of proinflammatory factors (Chen et al., 2019). Some studies have shown that the anti-inflammatory mechanism of GRg3 is related to the induction of M1 and M2 polarized macrophages and microglia (Im, 2020). However, our results provide another explanation for the anti-inflammatory mechanism of GRg3. In particular, administration of GRg3 significantly down-regulated the levels of mRNA and protein expression of *TLR4*, *MyD88*, and *NF-κB* p65. In addition, the levels of the inflammatory factor IL-1β decreased, whereas those of the downstream target, IL-10, increased. This finding suggests that GRg3 exerts an anti-inflammatory effect by restraining the TLR4 pathway. Importantly, the anti-inflammatory effect of GRg3 was shown to occur in a dose-dependent manner, with the highest dose exhibiting the strongest effect in regulating inflammation.

### 4.2 GRg3 effectively regulates intestinal flora

The intestinal flora interacts with intestinal epithelial cells to maintain stability of function of the intestinal barrier (Zaki et al., 2010; Brown et al., 2015; Li et al., 2020). Saponins (the main component of ginsenosides) inhibit intestinal inflammation, promote the repair of the intestinal barrier, maintain the diversity of the intestinal flora, and repair damaged colon tissues (Dong et al., 2019). Imbalances in the composition of the intestinal flora have been shown to be closely related to many inflammatory diseases, such as chronic colitis and Crohn’s disease (Mottawea et al., 2016; Gevers et al., 2017; Li et al., 2020). The intestinal tract is highly sensitive to IR (Gonzalez-Mercado et al., 2021). Oxygen, nitrogen, and other free radicals produced by IR further invade the intestinal mucosal epithelium (Sokol and Adolph, 2018). Concomitantly, IR can also disrupt the balance of the intestinal flora, significantly reducing its abundance and diversity, destroying tight junction proteins, and hence affecting the function of the intestinal barrier (Gerassy-Vainberg et al., 2018; Wang et al., 2019; Zhao et al., 2020; Li et al., 2020; Gonzalez-Mercado et al., 2021). Therefore, an imbalance in intestinal flora can aggravate RE (Darwich et al., 2014). Our results further confirmed that the number, richness, and diversity of intestinal microflora in ARP rats were significantly lower than those in normal rats, indicating a strong correlation between intestinal flora imbalance and ARP. After GRg3 treatment, the composition and distribution of intestinal flora significantly changed; the numbers of *Ruminococcus*, *Lactobacillus*, *Dubosiella*, *Blautia*, and *Romboutsia* organisms, as well as those of other beneficial bacteria, significantly increased. The distribution of these bacterial genera, except for *Ruminococcus*, was reduced in rats treated with dexamethasone. This indicates that, although dexamethasone maintained the diversity of intestinal flora, it restored the distribution only of *Ruminococcus* and did not significantly affect the numbers of other beneficial bacteria. In contrast, GRg3 regulated most of the beneficial bacteria in the gut, restoring the distribution of bacteria in the gut of ARP rats to levels closer to those found in normal rats. Therefore, we believe that GRg3 has more advantages than hormonal drugs in reshaping the intestinal flora and might replace other traditional drugs in the treatment of ARP in the future. Further analysis of the effects of different doses of GRg3 on different flora revealed that the numbers of beneficial bacteria were more significantly enriched in rats treated with high-dose GRg3, and *Ruminococcus*, *Lactobacillus*, and other beneficial bacteria might be used as biomarkers for the treatment of ARP. The intestinal flora can be regarded as an endocrine organ, with its metabolites being involved in the physiological function of the body. Imbalances in the composition of the intestinal flora lead to changes in the levels of a variety of metabolites, thus aggravating inflammatory damage and affecting intestinal function. Our functional gene analysis indicated that the administration of GRg3 was closely associated with enhanced cysteine and methionine metabolism. Therefore, further studies on GRg3 and amino acid metabolism are needed in the future.

In addition, we found that different doses of GRg3 resulted in different degrees of inflammatory relief, with high-dose GRg3 exhibiting the most significant effect. Although the final curative effect of the medium-dose GRg3 was similar to that of the high-dose GRg3, it took longer to have an effect, whereas low-dose GRg3 could not completely alleviate the inflammatory reaction in ARP rats. This finding is consistent with the results of our previous study, which showed that high-dose GRg3 has a significant curative effect in ARP rats (Hu and Cai, 2019).

## 5 Conclusion

This study demonstrated that GRg3 can regulate the TLR4/MyD88/NF-κB pathway, inhibit proinflammatory factors, effectively regulate intestinal flora, significantly increase the abundance of beneficial bacteria (such as *Ruminococcus* and *Lactobacillus*), and reduce inflammatory reactions. In addition to existing traditional treatments, Grg3 could be used as a new target for the treatment of ARP. However, the appropriate dosage regimen and side effects of GRg3 in the treatment of ARP were not covered in this study and need to be explored in future studies.

## Data availability statement

The data presented in the study are deposited in the NCBI BioProject repository, accession number PRJNA915595 and SRA repository, accession number SRP414737.

## Ethics statement

The animal study was reviewed and approved by the Medical Ethics Committee of Gansu Provincial Hospital.

## Author contributions

XD: methodology, writing-original draft, and software, HC: writing-review and editing, and conceptualization. TH: methodology and validation. LL: software and formal analysis. LZ: supervision and visualization. HW: formal analysis and data curation. LC: software. XL: supervision. All authors contributed to the article and approved the submitted version.
