# Supplementary material for: Ginsenoside Rg3 treats acute radiation proctitis through the TLR4/MyD88/NF-κB pathway and regulation of intestinal flora
Source: Front Cell Infect Microbiol. 2023 Jan 6;12:1028576. doi: 10.3389/fcimb.2022.1028576 (PMC9853003; doi:10.3389/fcimb.2022.1028576)
Supplement: Supplementary file 1 [file DataSheet_1.docx]

**Reagents**

Illumina NovaSeq sequencing platform (Nuohe Zhiyuan Company, Beijing, China).

ELISA kits (Faye Biological Corporation, Jiangsu, China)

TRIzol reagent, SYBR Green Master Mix (Yi Sheng Biological Company, Shanghai, China)

β-Actin, MyD88, and NF-κB P65 antibodies (Borges De Biological Company, Wuhan, China)

TLR4 and secondary antibodies (SAB Corporation, American)

Linear accelerator (Siemens, Germany)

Paraformaldehyde, RIPA, PMSF, phosphorylated protease inhibitor A and B solution, SDS-PAGE, PVDF, ECL chemiluminescent solution (Servicebio Company, Wuhan, China)

**TABLE**

| **Supplementary table 1 Disease activity index evaluation criteria** | | | |
| --- | --- | --- | --- |
| score | Loss of body weight(%) | Fecal character | Defecate haemorrhage |
| 0 | 0 | normal | normal |
| 1 | 1-5 | soft | occult blood positive |
| 2 | 6-10 | loose stool | occult blood strong positive |
| 3 | 11-15 | Mucoid stool | slightly bloody stool with naked eyes |
| 4 | 16-20 | Watery diarrhea | Naked eye bloody stool |

| **Supplementary table 2 The primers designed for RT-qPCR** | | | |
| --- | --- | --- | --- |
| Gene | | Sense primer | Antisense primer |
| TLR4 | CCAGGTGTGAAATTGAGACAATTG | | AAGCTGTCCAATATGGAAACCC |
| MyD88 | AAGGTGTCGTCGCATGGTG | | TTGGTGCAAGGGTTGGTATAGT |
| NF-κB | CAGATACCACTAAGACGCACCC | | CTCCAGGTCTCGCTTCTTCACA |
| GAPDH | CTGGAGAAACCTGCCAAGTATG | | GGTGGAAGAATGGGAGTTGCT |

**Supplementary figure**


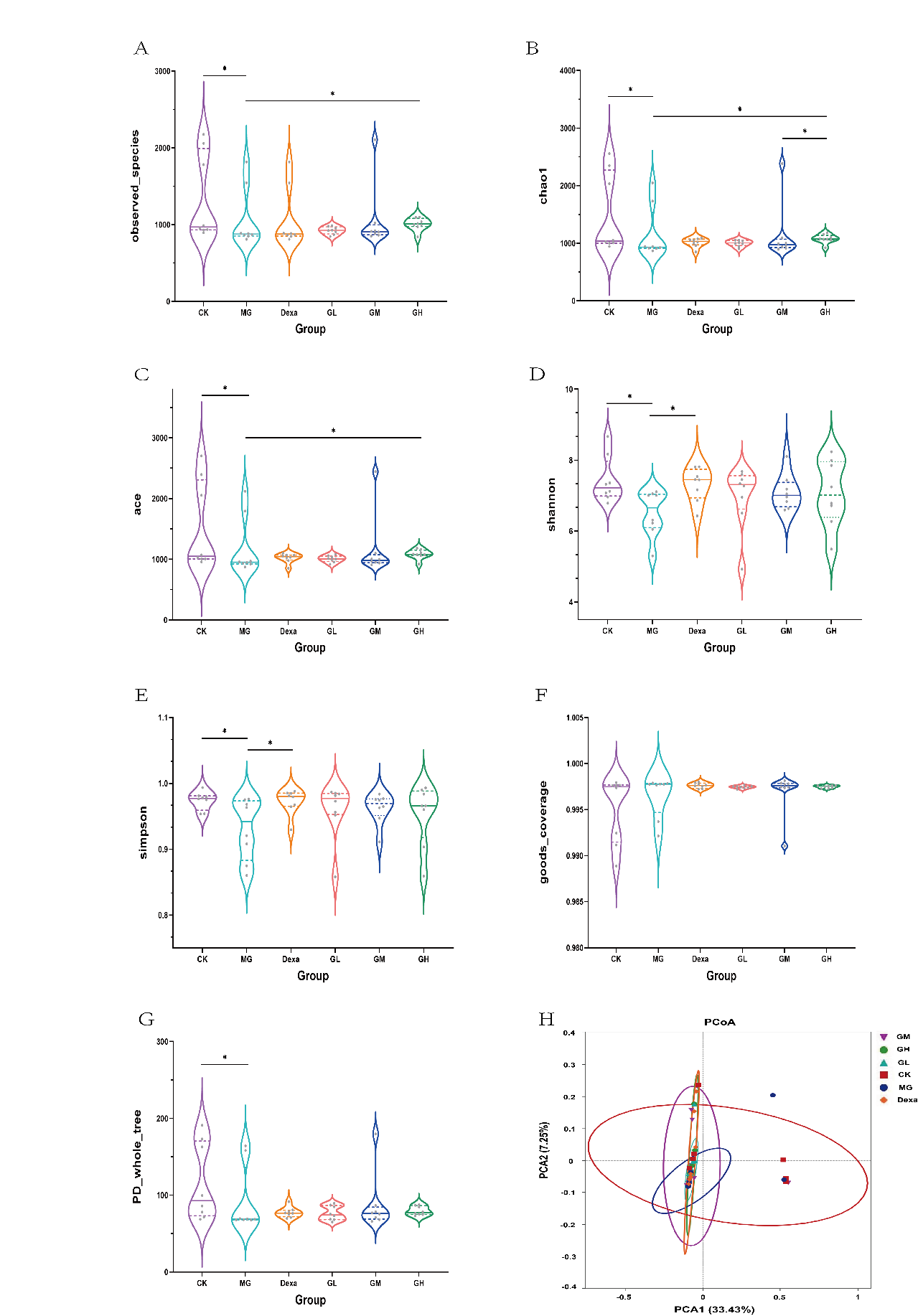


Supplementary figure 1 Alpha adversity and Beta adversity A-H: Observed species index, Chao1 index, ACE index, Shannon index, Simpson index, Goods coverage, PD whole tree, principal coordinate analysis (PcoA).


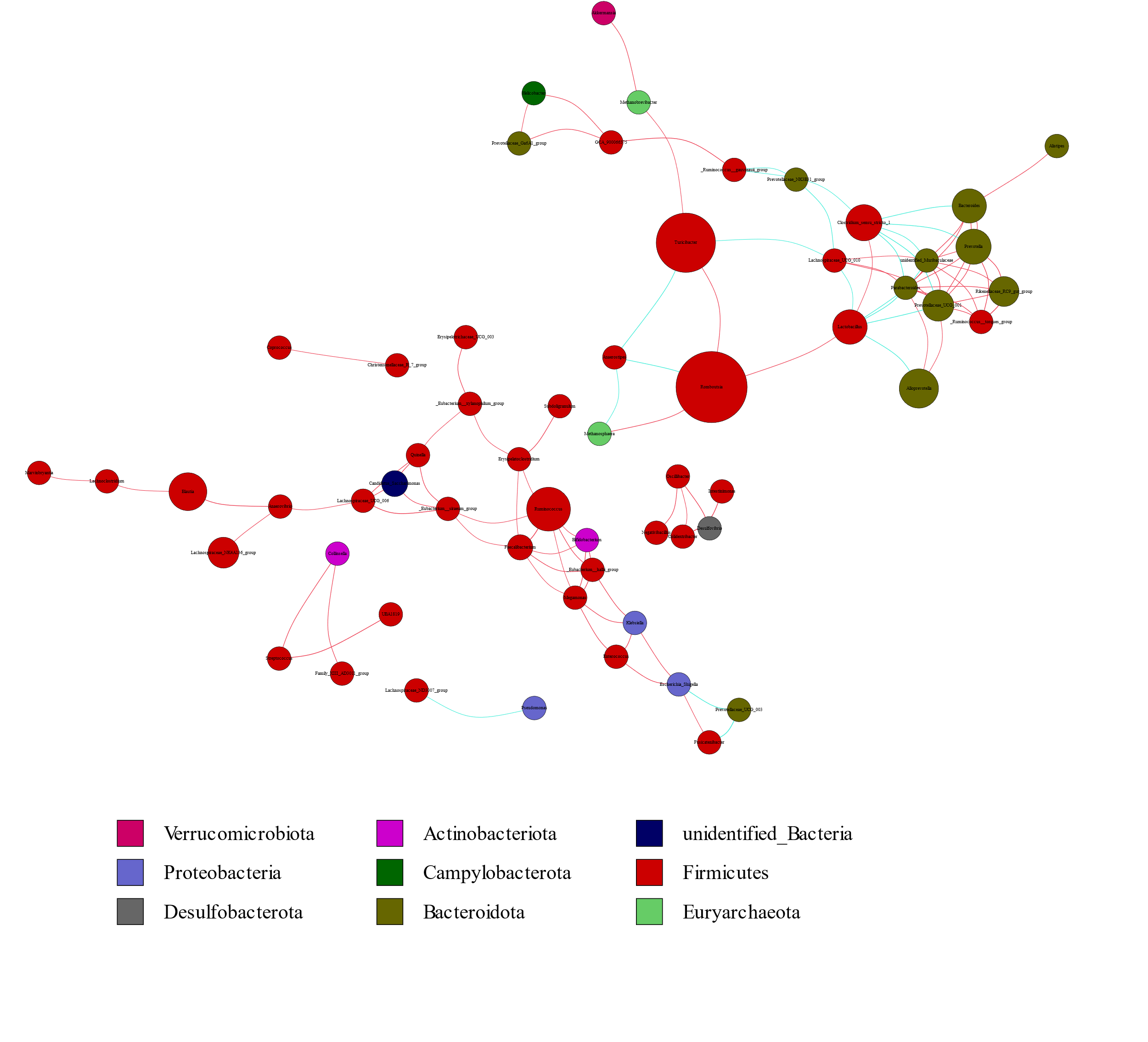


[..\..\..\..\..\..\Raw Data\16srRNA original data\05 network\spearman\network_samples_table_Samples_spearman p=0.01 阈值=0.6 物种数=100\network_samples_table_Samples_spearman_0.01_0.6_100.result\GH\genus.svg](../../../../../../Raw%20Data/16srRNA%20original%20data/05%20network/spearman/network_samples_table_Samples_spearman%20p=0.01%20阈值=0.6%20物种数=100/network_samples_table_Samples_spearman_0.01_0.6_100.result/GH/genus.svg)

Supplementary figure 2 In the species co-occurrence network map of GH. Different nodes represent different genera, node size represents the average relative abundance of this genus, nodes of the same gate have the same color, and there is a positive and negative correspondence between connection color and correlation (red positive correlation, blue negative correlation).


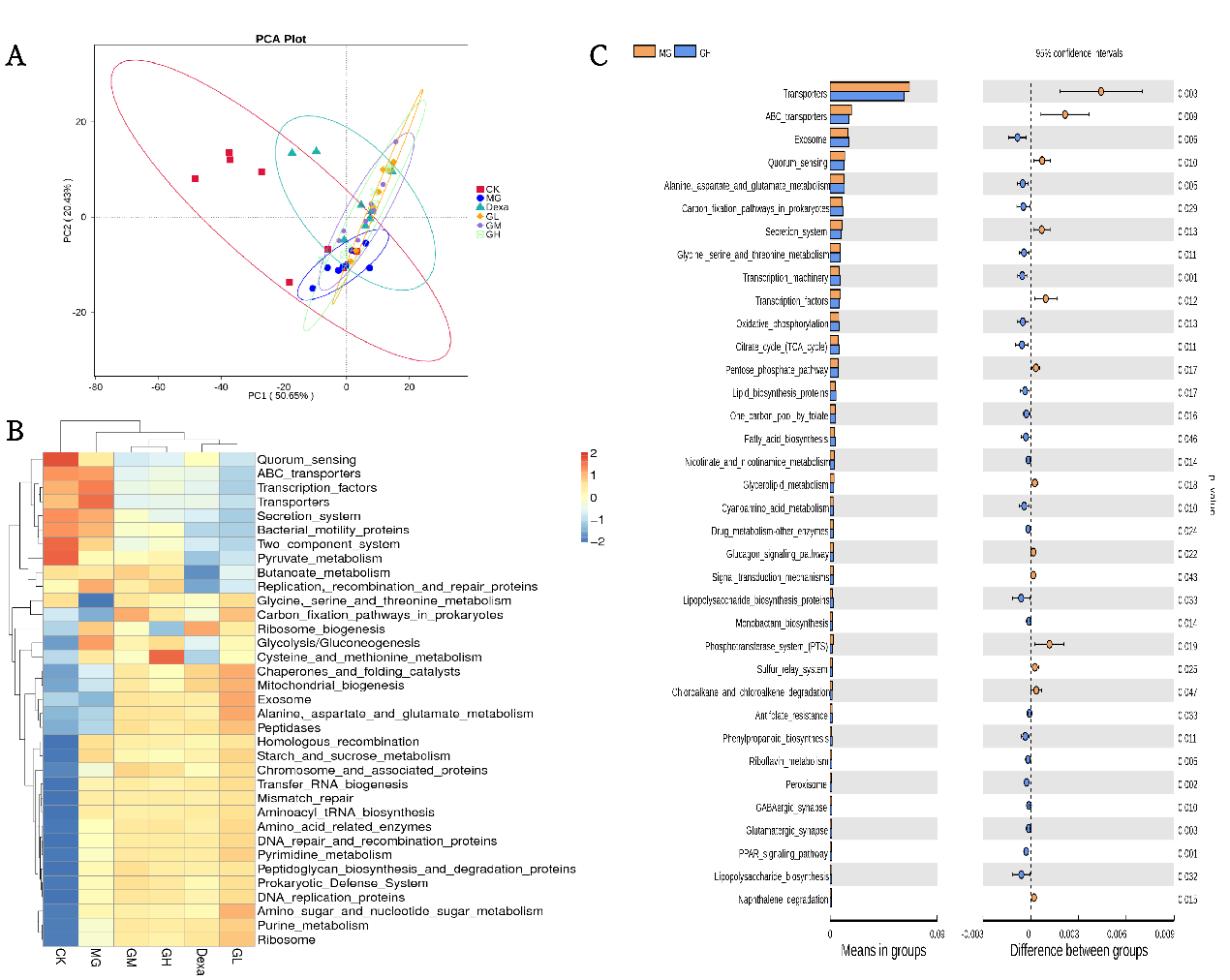


Supplementary figure 3 Tax4 Fun Gene function prediction A: PCA analysis; B: Level 3 functional relative abundance clustering heat map;C Analysis of the function of differential genes between the two groups by T-test.
